# Altered probe pressure and body position increase diagnostic accuracy for men and women in detecting hepatic steatosis using quantitative ultrasound

**DOI:** 10.1007/s00330-024-10655-1

**Published:** 2024-03-08

**Authors:** Marie Byenfeldt, Johan Kihlberg, Patrik Nasr, Christer Grönlund, Anna Lindam, Wolf C. Bartholomä, Peter Lundberg, Mattias Ekstedt

**Affiliations:** 1 Department of Radiology in Östersund, Östersund, Sweden; 2 Department of Radiation Science, Umeå University, Umeå, Sweden; 3 Department of Health, Medicine and Caring Sciences, Linköping University, Linköping, Sweden; 4 Center for Medical Image Science and Visualization, Linköping University, Linköping, Sweden; 5 Present Address: Department of Radiology in Linköping, Linköping, Sweden; 6 Division of Diagnostics and Specialist Medicine, Department of Health, Medicine and Caring Sciences, Linköping University, Linköping, Sweden; 7 Department of Public Health and Clinical Medicine, Umeå University, Umeå, Sweden; 8 Department of Radiation Physics, Linköping University, Linköping, Sweden; 9 Department of Medical and Health Science in Linköping University, Linköping, Sweden; 10 Center for Medical Image Science and Visualization, Linköping University, Linköping, Sweden

**Keywords:** Fatty liver, Ultrasonography, Magnetic resonance imaging, Sex factors, Patient positioning

## Abstract

**Objectives:**

To evaluate the diagnostic performance of ultrasound guided attenuation parameter (UGAP) for evaluating liver fat content with different probe forces and body positions, in relation to sex, and compared with proton density fat fraction (PDFF).

**Methods:**

We prospectively enrolled a metabolic dysfunction-associated steatotic liver disease (MASLD) cohort that underwent UGAP and PDFF in the autumn of 2022. Mean UGAP values were obtained in supine and 30° left decubitus body position with normal 4 N and increased 30 N probe force. The diagnostic performance was evaluated by the area under the receiver operating characteristic curve (AUC).

**Results:**

Among 60 individuals (mean age 52.9 years, SD 12.9; 30 men), we found the best diagnostic performance with increased probe force in 30° left decubitus position (AUC 0.90; 95% CI 0.82–0.98) with a cut-off of 0.58 dB/cm/MHz. For men, the best performance was in supine (AUC 0.91; 95% CI 0.81–1.00) with a cut-off of 0.60 dB/cm/MHz, and for women, 30° left decubitus position (AUC 0.93; 95% CI 0.83–1.00), with a cut-off 0.56 dB/cm/MHz, and increased 30 N probe force for both genders. No difference was in the mean UGAP value when altering body position. UGAP showed good to excellent intra-reproducibility (Intra-class correlation 0.872; 95% CI 0.794–0.921).

**Conclusion:**

UGAP provides excellent diagnostic performance to detect liver fat content in metabolic dysfunction-associated steatotic liver diseases, with good to excellent intra-reproducibility. Regardless of sex, the highest diagnostic accuracy is achieved with increased probe force with men in supine and women in 30° left decubitus position, yielding different cut-offs.

**Clinical relevance statement:**

The ultrasound method ultrasound-guided attenuation parameter shows excellent diagnostic accuracy and performs with good to excellent reproducibility. There is a possibility to alter body position and increase probe pressure, and different performances for men and women should be considered for the highest accuracy.

**Key Points:**

*• There is a possibility to alter body position when performing the ultrasound method ultrasound-guided attenuation parameter.*

*• Increase probe pressure for the highest accuracy.*

*• Different performances for men and women should be considered.*

**Graphical Abstract:**

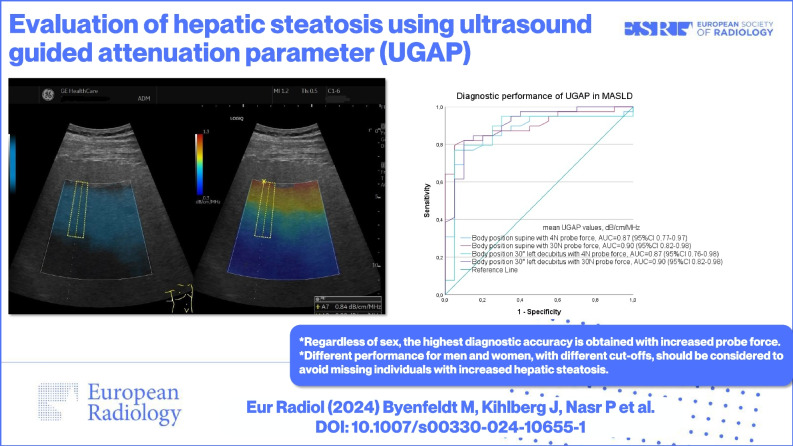

**Supplementary Information:**

The online version contains supplementary material available at 10.1007/s00330-024-10655-1.

## Introduction

Quantitative ultrasound (QUS) attenuation imaging of liver fat content can be difficult to perform depending on how the patient participates and their body constitution. How probe pressure and body position affect the diagnostic accuracy of QUS, especially in relation to sex, is currently unknown.

The estimated global prevalence of metabolic dysfunction-associated steatotic liver diseases (MASLD) is as high as 38% in some regions [1]. A recent nationwide adult cohort study in Sweden found a significantly higher hazard ratio for overall mortality in all individuals with MASLD, including simple steatosis, compared to controls [2].

The presence of hepatic steatosis is a prerequisite for diagnosing MASLD [3, 4]. Liver biopsy (LB) used to be the gold standard for determining liver fat content but has a risk of adverse events [5–8]. However, the proton density fat fraction (PDFF) determined by magnetic resonance imaging (MRI) is an excellent non-invasive method, surpassing and replacing LB as the reference method for hepatic fat quantification [9–12]. MRI has some limitations of accessibility and contraindications; therefore, conventional ultrasound (US) is recommended for diagnosing MASLD [13]. US uses a qualitative grading scale [14] with 85% sensitivity in grading S2–S3 [14, 15], but only 12% sensitivity in grading S0–S1 [16], as underlying liver diseases and low reproducibility reduce accuracy [17–19].

The US wave loses energy (i.e., attenuates) as it propagates in the tissue, and the attenuation can be quantitatively determined [20, 21]. All imaging modes in US start from radiofrequency (RF) data processing, and QUS for liver fat content is mostly based on RF data for calculation of the speed of sound, attenuation, and backscatter. Several studies have been performed for QUS determination of liver fat content with different technologies and US systems [22–32]. However, fat distribution differs between men and women [33], and reliability is important regardless of sex. To date, no standardized method for evaluating liver fat content by QUS has been published, and how increased probe pressure and body position affect diagnostic performance with the new technology US-guided attenuation parameter (UGAP) is not known [34].

The aims of the present study were to investigate the influence of probe pressure and body position on the diagnostic accuracy of UGAP in individuals with and without MASLD and to examine whether different thresholds are warranted for men and women for identifying the presence of hepatic steatosis (i.e., steatosis more than 5% according to MRI).

## Materials and methods

This prospective single-center study was approved by the Swedish Ethical Review Authority (No. 2022–01610-01). The research was performed in accordance with The World Medical Association Declaration of Helsinki, 2013. All study participants provided informed written consent. Data collection was performed between 24 September and 10 December 2022.

### Study population

**Fig. 1:**
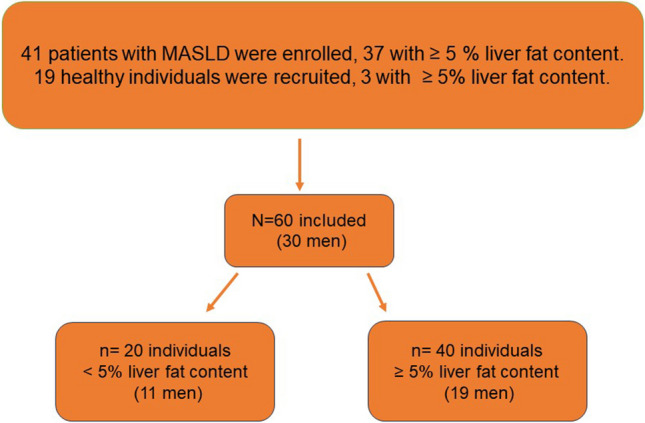
Flowchart of study population selection and distribution according to liver fat content measured by PDFF

Patients with a clinically verified diagnosis of MASLD were asked to participate in this study. Inclusion was based on the availability of a magnetic resonance examination including an MRI-PDFF > 10% performed within the last 12 months. A control population of healthy individuals with low risk of MASLD (i.e., BMI < 25 kg/m^2^ and absence of components of metabolic syndrome) were also asked to participate in this study. The study population quota was deemed met when 40 individuals with ≥ 5% liver fat content and 20 individuals with < 5% liver fat content according to PDFF had been included and equal distribution of sex was fulfilled in the whole study group. All patients were recruited at the Department of Gastroenterology and Hepatology, Linköping University Hospital. Exclusion criteria were declining to participate, age < 18 years, pregnancy, or contraindications to MRI (i.e., claustrophobia or presence of medical implants or implants with ferromagnetic properties). Thus, a total of 60 individuals were included after giving written informed consent (Fig. 1).

### Ultrasound measurements and performance

The US-guided attenuation parameter (UGAP) was measured using a LOGIQ E10 rev 3 US system (General Electric Healthcare) with a convex probe (C1-6). In the UGAP program, the US display was divided into two, with a quality map to select the best image for the attenuation coefficient measure and an attenuation map where attenuation is quantified and shown in a large area using a real-time color-coded map. With the UGAP technique, an entire measurement loop is automatically recorded from five measurements, skipping 10 frames between each measurement. Measurements were performed with a rectangular-shaped region of interest (ROI), the position of which could be altered only in the lateral direction in the liver parenchyma. Reliable UGAP measurements were defined as the median values of five measurements with a ratio of the interquartile range of the attenuation value to the median being < 30% and an approved quality map. Failure was defined as a red-marked ROI in the display attenuation map. UGAP values were expressed in dB/cm/MHz and obtained from a homogeneous area of liver parenchyma free of big vessels and rib shadow with the patient’s right arm elevated above their head.

Five UGAP measurements were performed with normal 4 Newton (N) or increased 30 N probe force and with the body in supine or 30° left decubitus position using two identical pillows to ensure an identical 30° position. For a description of applied probe force measurements, see Supplementary Material 1 (*Method description of applied probe force measurements*)*.*

To evaluate the reliability of UGAP, sixty measurements were performed in the supine position with normal 4 N probe force, and sampling was repeated approximately 1–3 months later with the same operator, blinded to the earlier results, from recorded volumes.

All UGAP measurements were obtained on the same day as PDFF and magnetic resonance elastography (MRE), and all operators were blinded to each method´s results. Prior to the examination, all participants were fasting for at least 5 h.

As steatosis in early stages commonly has heterogeneous distribution in the liver, there may be a need for increased exactness in the evaluation of the diagnostic performance of UGAP and the influence of probe force, which is provided in Supplementary Material 2 (*Evaluation of UGAP diagnostic performance with single measurement in same spot of liver parenchyma* and Supplementary Material 3, *Evaluation of probe force with single measurement in same spot of liver parenchyma*).

### Magnetic resonance measurements and performance

PDFF and MRE were performed using an Ingenia 3 T (Philips Healthcare, Best). The PDFF [35] covered the entire liver, and the MRE acquisitions were planned in four slices according to a standardized protocol [36]. Complete information is provided in Supplementary Material 4 (*Method description of magnetic resonance measurements*)*.*

### Statistical analysis

The normality of the continuous data for UGAP and MRI-PDFF was tested by the one-sample Kolmogorov–Smirnov test.

Intra-class correlations (ICCs) were estimated for UGAP, and their 95% confidence intervals (CIs) were based on a mean-rating (k = 2), absolute-agreement, and 2-way mixed-effects model. ICC values < 0.50 indicate poor reliability, values between 0.50 and 0.75 indicate moderate reliability, values between 0.75 and 0.90 indicate good reliability, and values > 0.90 indicate excellent reliability [37].

The paired t-test was used to evaluate differences between mean UGAP values in different body positions and different probe forces.

To evaluate the diagnostic accuracy, ROC analysis was performed. From the area under the receiver operating characteristic curve (AUC), a cut-off was determined for the mean UGAP value at sensitivity > 90% and 95% and specificity > 90% and 95%. We used an MRI-PDFF cut-off of 5% to define UGAP diagnostic performance for distinguishing steatosis grade S0 vs. ≥ S1. The evaluation was made in different body positions and probe forces and in subgroups of men and women.

The corresponding levels of MRI-PDFF for the different grades of steatosis were set in accordance with Imajo et al [30]: steatosis grade 1 (S1) = 5.0–11.2%, S2 = 11.3–17.0%, and S3 ≥ 17.1%.

The corresponding levels of MRE for the different grades of fibrosis were set in accordance with Guglielmo et al [38]: normal =  < 2.9 kPa, fibrosis stage 1 = 2.9–3.5 kPa, fibrosis stage 2 = 3.5–4.0, fibrosis stage 3 = 4.0–5.0 kPa, and fibrosis stage 4 or cirrhosis =  > 5.0 kPa.

Continuous variables are expressed as the mean ± standard deviation (min–max). Categorical variables are expressed as frequency and percentage.

For all statistical tests, 95% CIs were calculated. A *p* value < 0.05 was considered significant, and all statistical tests were two-sided.

Statistical analyses were performed in the software Statistical Package for Social Sciences (SPSS) version 29.

## Results

No technical failure occurred in the performance of technical measures of UGAP. A total of 60 individuals (30 men, mean age 52.9 years ± 12.9 and 30 women, 53.9 years ± 11.6) were included in the analysis, including 40 individuals with MASLD and 20 control individuals without MASLD. The characteristics of all participants in the study, stratified by sex, are presented in Table 1.

**Table 1:** Characteristics of the study population

	Men (*n* = 30)	Women (*n* = 30)	*p* value
**(*N*** **= 60)**			
**Age mean years (SD, range)**	52.87 (12.86; 31–74)	53.90 (11.56; 32–75)	0.745
non-MASLD	47.18 (12.60)	47.22 (11.67)	0.994
MASLD	56.16 (12.12)	56.76 (10.51)	0.867
**Body mass index, kg/m**^**2**^	28.62 (4.97)	28.73 (6.00)	0.934
non-MASLD	25.81 (5.34)	23.89 (3.57)	0.369
MASLD	30.24 (4.05)	30.81 (5.64)	0.718
**Waist to hip ratio**	0.99 (0.07)	0.91 (0.08)	< 0.001
non-MASLD	0.96 (0.08)	0.82 (0.05)	< 0.001
MASLD	1.02 (0.06)	0.94 (0.06)	< 0.001
**Sagittal abdominal diameter, cm**	23.13 (3.99)	21.73 (4.96)	0.233
non-MASLD	20.26 (3.35)	17.00 (1.90)	0.018
MASLD	24.80 (3.38)	23.76 (4.45)	0.416
Skin-to-liver capsule distance*, cm (range)	2.20 (1.42–3.19)	1.96 (0.94–3.13)	0.061
non-MASLD	1.92 (1.42–3.19)	1.43 (0.94–1.98)	0.032
MASLD	2.36 (1.69–3.09)	2.18 (1.59–3.13)	0.131

Values are mean (SD) or mean (min–max) unless otherwise noted. *p*-value derived from t-test between values for men and women. *MASLD* metabolic dysfunction-associated steatotic liver disease (defined as ≥ 5% MRI-PDFF), *MRI* magnetic resonance imaging, *PDFF* proton density fat fraction, *Skin-to-liver capsule distance for body in supine position with normal 4 N probe

### Reliability of UGAP

Sixty measurements performed with the body in the supine position with normal 4 N probe force yielded a mean UGAP value of 0.65 ± 0.14 dB/cm/MHz. Approximately 1–3 months later, the same operator, blinded to the earlier results, obtained 60 measurements from recorded volumes, yielding a mean UGAP value of 0.66 ± 0.12 dB/cm/MHz. There was no significant difference in UGAP values between the two time points (*p* = 0.38, *N* = 60), and the estimated ICC was 0.872 (95% CI 0.794–0.921). Based on the ICC, the intra-rater and test–retest reliability of this new method shows good to excellent reliability.

### UGAP performance for staging hepatic steatosis in MASLD

**Fig. 2:**
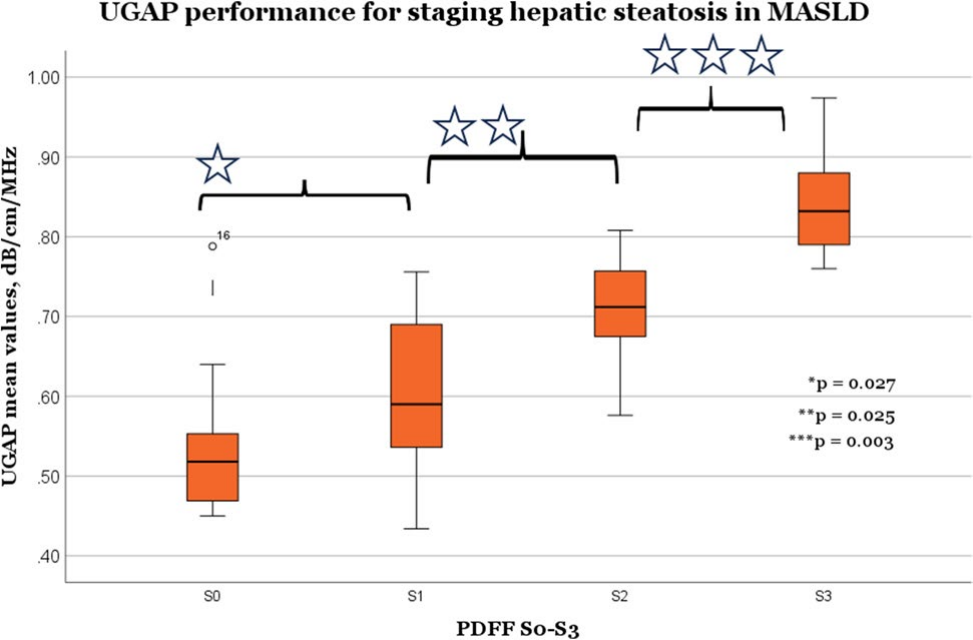
UGAP diagnostic performance for staging hepatic steatosis in MASLD. There was a significant difference between hepatic steatosis stages S0-S3 in the supine position with normal 4 N probe force. S0, *n* = 20; S1, *n* = 18; S2, *n* = 8; S3, *n* = 14. Differences in the mean UGAP values: S0 0.53 vs. S1 0.61 dB/cm/MHz (*p* = 0.027), S1 0.61 vs. S2 0.71 dB/cm/MHz (*p* = 0.025), S2 0.71 vs. S3 0.84 dB/cm/MHz (*p* = 0.003). MASLD= metabolic dysfunction-associated steatotic liver disease (defined as ≥ 5% MRI-PDFF), UGAP = ultrasound-guided attenuation parameter, MRI = magnetic resonance imaging, PDFF = proton density fat fraction. PDFF defined cut-offs for steatosis grade 1 (S1) = 5.0–11.2%, S2 = 11.3–17.0%, and S3 ≥ 17.1%

There was a significant difference between hepatic steatosis stages S0–S3 in the supine position with normal 4 N probe force (Fig. 2). Differences in the mean UGAP values were S0 0.53 vs. S1 0.61 dB/cm/MHz (*p* = 0.027, *n* = 20; *n* = 18), S1 0.61 vs. S2 0.71 dB/cm/MHz (*p* = 0.025, *n* = 18; *n* = 8), S2 0.71 vs. S3 0.84 dB/cm/MHz (*p* = 0.003, *n* = 8; *n* = 14).

### Influence of probe force and body position in UGAP

For the whole group in a supine position, there was a significant difference between the mean UGAP values obtained with normal 4 N and increased 30 N probe force (*p* < 0.001, *N* = 60). However, there was no significant difference with the body in the 30° left decubitus position (*p* = 0.195, *n* = 59). For men, there was no difference in the supine (*p* = 0.093, *n* = 30) or 30° left decubitus position (*p* = 0.104, *n* = 29). For women, there was a difference in the supine position (*p* = 0.004, *n* = 30), but not in the 30° left decubitus position (*p* = 0.104, *n* = 30; Table 2).

**Table 2:** Differences in mean UGAP values with different probe forces performed in different body positions

UGAP performance with different probe force in MASLD	Normal probe force, 4 N	Increased probe force, 30 N	*p* value*
	Mean dB/cm/MHz (SD)	Mean dB/cm/MHz (SD)	
**Body in supine position**			
**All,** ***N*** **= 60**	0.65 (0.14)	0.68 (0.13)	0.001
Women, ***n*** = 30	0.63 (0.14)	0.66 (0.12)	0.004
Men, ***n*** = 30	0.67 (0.14)	0.69 (0.14)	0.093
**Body in 30° left decubitus position**			
**All,** ***n*** = 59**	0.65 (0.14)	0.66 (0.13)	0.195
Women, ***n*** = 30	0.65 (0.13)	0.65 (0.12)	0.794
Men, ***n*** = 29	0.65 (0.14)	0.67 (0.13)	0.104

*Paired t-test. **Missing data for one participant in 30° left decubitus body position. *UGAP* ultrasound-guided attenuation parameter, *MASLD* metabolic dysfunction-associated steatotic liver disease (defined as ≥ 5% MRI-PDFF)

No significant differences were found for the mean UGAP values in different body positions (Table 3).

**Table 3:** Mean UGAP values in different body positions with different probe forces

UGAP performed in different body positions in MASLD	Body in supine position	Body in 30° left decubitus position	*p* value*
	Mean dB/cm/MHz (SD)	Mean dB/cm/MHz (SD)	
**Normal probe force, 4 N**			
**All,** ***n*** = 59**	0.64 (0.14)	0.65 (0.15)	0.501
Women, ***n*** = 30	0.63 (0.14)	0.65 (0.13)	0.110
Men, ***n*** = 29	0.66 (0.14)	0.65 (0.14)	0.610
**Increased probe force, 30 N**			
**All,** ***n*** = 59**	0.67 (0.13)	0.66 (0.12)	0.224
Women, ***n*** = 30	0.66 (0.12)	0.65 (0.12)	0.518
Men, ***n*** = 29	0.68 (0.14)	0.67 (0.13)	0.287

*Paired t-test. **Missing data for one participant in 30° left decubitus body position. *UGAP* ultrasound-guided attenuation parameter, *MASLD* metabolic dysfunction-associated steatotic liver disease (defined as ≥ 5% MRI-PDFF)

Increased probe force showed a significant impact on skin-to-liver capsule distance; complete results are provided in Supplementary Material 5 (*Results for probe force influence on skin-to-liver capsule distance*).

### Validity of UGAP in MASLD 

**Fig. 3:**
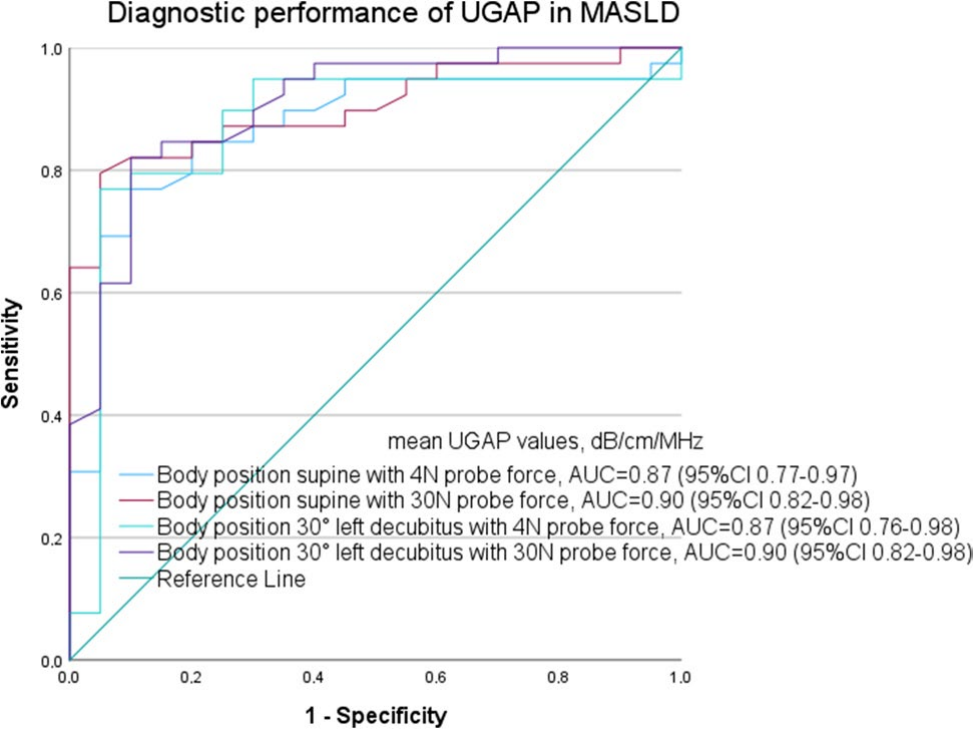
AUC plots illustrating the diagnostic performance of the ultrasound-guided attenuation parameter (UGAP) for the body in a supine position with normal 4 N probe force, yielding an AUC of 0.87 (95% CI 0.77–0.97), and with increased 30 N probe force (AUC 0.90, 95% CI 0.82–0.98). Body in 30° left decubitus body position with normal 4 N probe force yielded an AUC of 0.87 (95% CI 0.76–0.98), and with increased 30 N probe force an AUC of 0.90 (95% CI 0.82–0.98). MRI-PDFF ≥ 5% was defined as the cut-off for steatosis grade S1. CI = confidence interval, AUC = area under the curve, MASLD= metabolic dysfunction-associated steatotic liver disease (defined as ≥ 5% MRI-PDFF), MRI = magnetic resonance imaging, PDFF = proton density fat fraction

For the whole group, the best diagnostic performance of UGAP was found with the body in the 30° left decubitus position with increased 30 N probe force (AUC 0.90, 95% CI 0.82–0.98) with a cut-off of 0.58 dB/cm/MHz, 90% sensitivity, and 70% specificity (Fig. 3 and Table 4).

**Table 4:** Probe force and body position influence on the diagnostic performance of UGAP in MASLD

Diagnostic performance for UGAP in MASLD, *N* = 60	AUC (95%CI)	Set sensitivity or specificity level	UGAP mean value dB/cm/MHz	Sensitivity, %	Specificity, %
***Body in supine position***					
Normal probe force, 4 N	0.87 (0.77–0.97)	Sensitivity 90%	0.53	**90**	60
		Sensitivity 95%	0.52	**95**	50
		Specificity 90%	0.59	77	**90**
		Specificity 95%	0.64	69	**95**
Increased probe force, 30 N	0.90 (0.82–0.98)	Sensitivity 90%	0.55	**90**	50
		Sensitivity 95%	0.54	**95**	45
		Specificity 90%	0.62	82	**90**
		Specificity 95%	0.64	80	**95**
* Women n* = *30*					
Normal probe force, 4 N	0.93 (0.82–1.00)	Sensitivity 90%	0.53	**90**	78
		Sensitivity 95%	0.52	**95**	78
		Specificity 90%	0.54	86	**90**
		Specificity 95%	0.55	81	**95**
Increased probe force, 30 N	0.90 (0.79–1.00)	Sensitivity 90%	0.56	**90**	66
		Sensitivity 95%	0.54	**95**	56
		Specificity 90%	0.62	76	**90**
		Specificity 95%	0.65	0.76	**95**
* Men n* = *30*					
Normal probe force, 4 N	0.83 (0.67–0.99)	Sensitivity 90%	0.57	**90**	64
		Sensitivity 95%	0.53	**95**	46
		Specificity 90%	0.66	78	**90**
		Specificity 95%	0.78	28	**95**
Increased probe force, 30 N	0.91 (0.81–1.00)	Sensitivity 90%	0.60	**90**	82
		Sensitivity 95%	0.54	**95**	36
		Specificity 90%	0.62	89	**90**
		Specificity 95%	0.69	78	**95**
***Body in 30° left decubitus position***					
Normal probe force, 4 N	0.87 (0.76–0.98)	Sensitivity 90%	0.57	**90**	75
		Sensitivity 95%	0.55	**95**	70
		Specificity 90%	0.61	80	**90**
		Specificity 95%	0.62	77	**95**
Increased probe force, 30 N	0.90 (0.82–0.98)	Sensitivity 90%	0.58	**90**	70
		Sensitivity 95%	0.55	**95**	65
		Specificity 90%	0.63	82	**90**
		Specificity 95%	0.69	62	**95**
* Women n* = *30*					
Normal probe force, 4 N	0.88 (0.76–1.00)	Sensitivity 90%	0.57	**90**	67
		Sensitivity 95%	0.55	**95**	67
		Specificity 90%	0.61	76	**90**
		Specificity 95%	0.62	71	**95**
Increased probe force, 30 N	0.93 (0.83–1.00)	Sensitivity 90%	0.58	**90**	78
		Sensitivity 95%	0.56	**95**	78
		Specificity 90%	0.62	81	**90**
		Specificity 95%	0.68	57	**95**
* Men n* = 29*					
Normal probe force, 4 N	0.85 (0.68–1.00)	Sensitivity 90%	0.58	**90**	82
		Sensitivity 95%	0.52	**95**	64
		Specificity 90%	0.62	83	**90**
		Specificity 95%	0.67	61	**95**
Increased probe force, 30 N	0.89 (0.77–1.00)	Sensitivity 90%	0.64	**90**	90
		Sensitivity 95%	0.54	**95**	46
		Specificity 90%	0.64	90	**90**
		Specificity 95%	0.76	50	**95**

Steatosis grade ≥ S1 defined as MRI-PDFF ≥ 5%. *Missing data for 1 participant in 30° left decubitus position. *AUC* area under the curve, *CI* confidence interval, *MASLD* metabolic dysfunction-associated steatotic liver disease (defined as ≥ 5% MRI-PDFF), *UGAP* ultrasound-guided attenuation parameter, *MRI* magnetic resonance imaging, *PDFF* proton density fat fraction

**Fig. 4:**
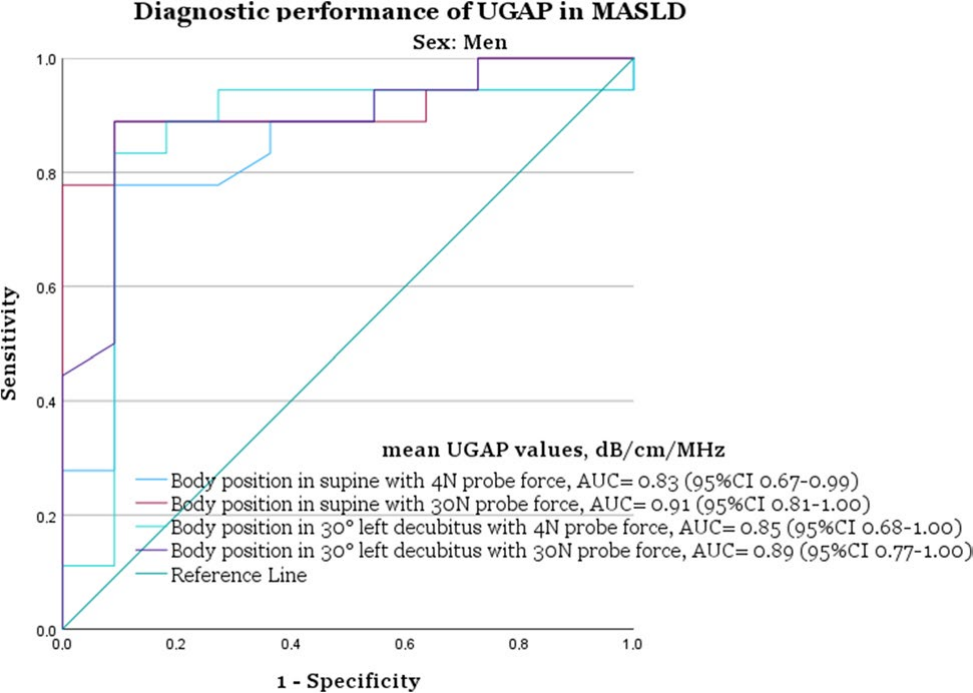
AUC plots illustrating the diagnostic performance of ultrasound-guided attenuation parameter (UGAP) for men (*n* = 30). UGAP was performed with normal 4 N force, increased 30 N probe force, and with the body in the supine or 30° left decubitus position. MRI-PDFF < 5% was the cut-off for steatosis grade S0 (*n* = 11) and ≥ S1 (*n* = 19). With the body in the supine position and with normal 4 N probe force had an AUC of 0.83 (95% CI 0.67–0.99), and with increased 30 N probe force an AUC of 0.91 (95% CI 0.81–1.00). With the body in 30° left decubitus position and with normal 4 N probe force, the AUC was 0.85 (95% CI 0.68–1.00), and with increased 30 N probe force 0.89 (95% CI 0.77–1.00). AUC = area under the curve, CI = confidence interval, MASLD= metabolic dysfunction-associated steatotic liver disease (defined as ≥ 5% MRI-PDFF), MRI = magnetic resonance imaging, PDFF = proton density fat fraction

**Fig. 5:**
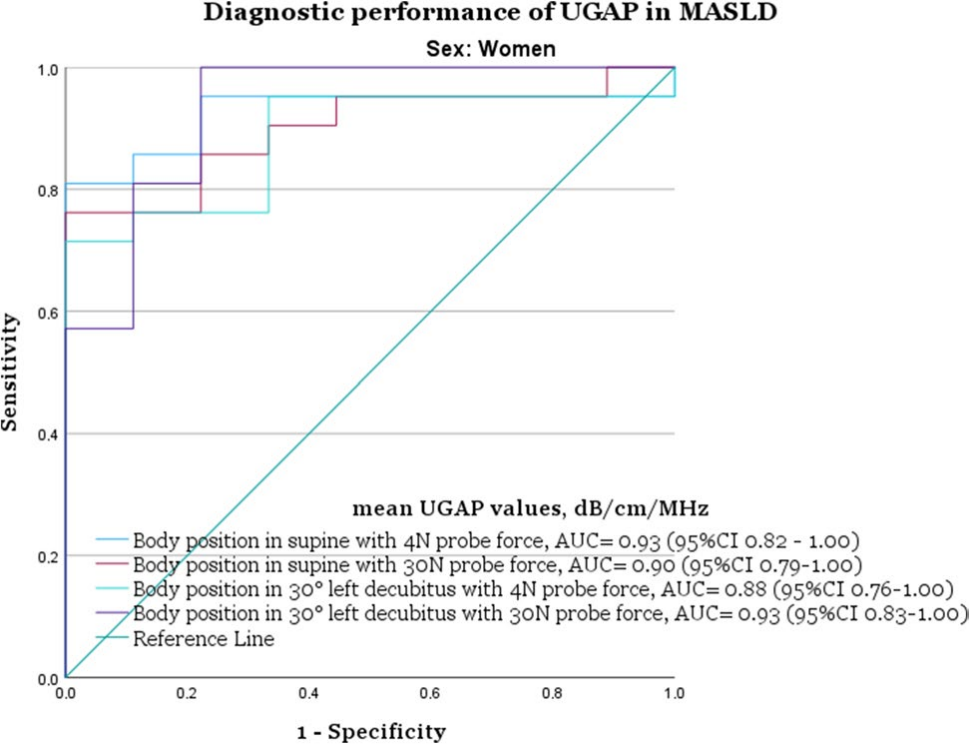
AUC plots illustrating the diagnostic performance of ultrasound-guided attenuation parameter (UGAP) for women (*n* = 30). UGAP was performed with normal 4 N probe force, increased 30 N probe force, and with the body in the supine or 30° left decubitus position. MRI-PDFF < 5% was the cut-off for steatosis grade S0 (*n* = 11) and ≥ S1 (*n* = 19). The supine position with normal 4 N probe force had an AUC of 0.93 (95% CI 0.82–1.00) and with increased 30 N probe force 0.90 (95% CI 0.79–1.00). With the body in 30° left decubitus position with normal 4 N probe force, the AUC was 0.88 (95% CI 0.76–1.00), and with increased 30 N probe force 0.93 (95% CI 0.83–1.00). AUC = area under the curve, CI = confidence interval, MASLD= metabolic dysfunction-associated steatotic liver disease (defined as ≥ 5% MRI-PDFF), MRI = magnetic resonance imaging, PDFF = proton density fat fraction

In the group of men, the best performance was in the supine position with increased 30 N probe force (AUC 0.91, 95% CI 0.80–1.00), with a cut-off of 0.60 dB/cm/MHz, sensitivity of 90%, and specificity of 82%. For women, the best performance was in the 30° left decubitus position with increased 30 N probe force (AUC 0.93, (95% CI 0.82–1.00), with a cut-off of 0.56 dB/cm/MHz, sensitivity of 95%, and specificity of 78% (Figs. 4 and 5 and Table 4).

With normal 4 N probe force, the best diagnostic accuracy for men was with the body in the 30° left decubitus position (AUC 0.85, 95% CI 0.68–1.00), with a cut-off of 0.58 dB/cm/MHz, 90% sensitivity, and 82% specificity. For women, the best diagnostic accuracy was with the body in the supine position (AUC 0.93, 95% CI 0.82–1.00), with a cut-off of 0.52 dB/cm/MHz, 95% sensitivity, and 78% specificity (Figs. 4 and 5 and Table 4).

### MRI PDFF and MRE measurement results

Using Imajio et al PDFF defined cut-offs for liver steatosis grade S0–S3 yielded in 20 participants with S0, 18 participants with S1, 8 participants with S2, and 14 participants with S3.

Using Guglielmo et al defined cut-offs for liver fibrosis grades 0–4 yielded in 58 participants with no liver fibrosis (< 2.9 kPa), 1 participant with liver fibrosis stages 1–2 (2.9–3.5 kPa) and steatosis grade S3, and 1 participant with liver fibrosis stages 2–3 (3.5–4.0 kPa) and steatosis grade S2.

## Discussion

We assessed 60 individuals using the new QUS method UGAP to stage liver fat content in different body positions and with different probe forces and assessed men and women separately to detect potential sex differences. The results showed that an increased 30 N probe force yielded increased mean UGAP values and higher diagnostic accuracy compared to normal 4 N probe force, body position did not influence the mean UGAP values, and differences between sexes indicated different examination techniques for men and women.

To the best of our knowledge, no study has been conducted with increased probe force or to evaluate the differences between men and women examined by QUS for liver fat content. Consistent with our results, earlier studies of UGAP have shown good inter-reproducibility [39] and no correlation with liver fibrosis [40, 41].

An intercostal approach in the supine position with normal probe force is recommended for the estimation of liver fat content by US attenuation [42]. The distance to the liver increases in obesity and overweight, and the B-mode image of US is known to be affected during these conditions. Therefore, altering body position and increasing probe pressure are common techniques to obtain acceptable B-mode images in abdominal US examinations. However, the possibility of using these examination techniques for UGAP has not yet been evaluated. In the present study, we showed that the body positions from supine to 30° left decubitus are interchangeable, and higher mean UGAP values were obtained with increased 30 N probe force, though the best accuracy was obtained with higher probe force.

There are well-known differences between the sexes in how body fat is distributed; women have a greater ability to store fat in subcutaneous sections, whereas men have more visceral fat than women [43, 44], which may indicate a need for different examination techniques for men and women to achieve higher diagnostic accuracy. In the present study, the best diagnostic accuracy for women was obtained with the body in the 30° left decubitus position with increased 30 N probe force and for men with the body in the supine position with increased 30 N probe force. Accordingly, our results suggest that the operator should consider the patient’s sex when performing UGAP measurements and consider the use of different cut-offs for women and men. Interpreting our results in clinical settings may result in reclassification of the diagnosis.

The impact on the reliability of QUS due to increased distance to the liver has been confirmed previously [45]. Increased probe pressure induces tissue deformation in the subcutaneous layers between the probe and costal rib, and the increased 30 N probe force in our study decreased the skin-to-liver capsule distance. In comparison, in QUS shear wave elastography for liver fibrosis, the use of increased probe pressure yielded improved technical success with more feasible investigations [46]. The positive effect of increased probe force can be explained by a reduced impact of scattering, refraction, absorption, and reflection in the boundary region, which enables the US waves to better traverse in compressed subcutaneous fatty layers than non-compressed layers between the skin and costal rib, with less energy loss. This hypothesis is consistent with earlier findings implying that the QUS shear wave method is affected by tissue attenuation [47]. Also, increased skin-to-liver capsule distance has higher amounts of aberrations in the RF signal which might have a negative impact on QUS attenuation estimation, and has been earlier discussed as a possible confounding factor [31].

In our study, we determined the attenuation coefficient in an MASLD cohort with equal numbers of men and women, which is a strength. Liver fat content was determined with PDFF as the reference method for all participants, which also is a strength, and UGAP and MRI measurements were performed on the same day for each participant. Moreover, this study was performed with UGAP rev 3 in US device GE E10 LOGIQ and defined PDFF steatosis stages were similar to previously published data, which ensures the possibility of reliable comparisons [30]. In our study, 58 participants did not have fibrosis according to our MRE measurements. Only 1 participant with liver fibrosis stages 1–2 (2.9–3.5 kPa) and steatosis grade S3, and 1 participant with liver fibrosis stages 2–3 (3.5–4.0 kPa) and steatosis grade S2. It is known that with increasing levels of fibrosis, the grade of steatosis declines over time, and can therefore be seen as a confounding factor in determining the presence of steatosis. Therefore, another strength in our study is the evaluation of fibrosis performed with MRE which is valuable for comparison with other similar research. It is also valuable since we do not know the full impact of liver fibrosis on hepatic steatosis measurements with UGAP.

This work has some limitations. The clinical usefulness of our results may be limited due to a lack of available commercial probe pressure devices, and a technical solution for recording the probe pressure was previously requested for QUS [48]. However, we showed that as much as 30 N of probe force does not compromise the UGAP attenuation coefficient results, but indicated that increased probe force is beneficial for increased diagnostic accuracy. Moreover, only one operator performed UGAP. Error sources, such as backscattering variation, speed of sound variation, focus location, imaging artifacts, image resolution, and signal-to-noise ratio [42], can increase intra-inter operator variability, which may impact the benefits of increased probe force for diagnostic accuracy. The differences found in our study between men and women should also be evaluated in a larger cohort to confirm the need for different performance and cut-offs for men and women for the highest diagnostic accuracy. Another limitation is the lack of liver biopsy used as reference for hepatic steatosis stages S2 and S3 in this study; however, the cut-off to detect hepatic steatosis ≥ S1 used in this study was 5%.

There are several commercial QUS methods available for measuring liver fat content, though not all possible confounders have been evaluated [31, 32, 49]. Moreover, in medical imaging, different patterns of hepatic steatosis have been described (diffuse, geographic, focal, subcapsular, multifocal, perivascular) [50, 51]. In cases of geographic pattern, with UGAP, there is the possibility to alter the ROI in lateral directions to select parts in the liver parenchyma with increased fat content, but how to perform measurements with QUS in these cases has not yet been discussed. In UGAP the ROI size and position in depth are fixed, due to the integrated reference phantom, which seems to be beneficial compared to other technologies in which high variability is seen for attenuation estimates depending on ROI depth [26]. In lower steatosis stages, the distribution of steatosis is known to be heterogenous, which has an impact on diagnostic accuracy. However, with single measurements and small PDFF ROIs, we showed excellent diagnostic performance for UGAP, Supplementary Material 2, (*Evaluation of UGAP diagnostic performance with single measurement in same spot of liver parenchyma*). A standardized method is needed for the estimation of liver fat content by QUS attenuation.

## Conclusion

The UGAP provides excellent diagnostic performance to detect ≥ S1 in MASLD (defined as MRI-PDFF 5%) and has good to excellent reproducibility. Altering body position to 30° left decubitus is possible when performing UGAP, and increased 30 N probe force results in increased mean UGAP values. Regardless of sex, the highest diagnostic accuracy is obtained with increased probe force, with men in the supine and women in the 30° left decubitus position, rendering different cut-offs, which should be considered to avoid missing individuals with increased hepatic steatosis. We therefore conclude that both probe force and body position influence the accuracy of UGAP in individuals with MASLD and that different thresholds should be used for men and women.

### Supplementary Information

Below is the link to the electronic supplementary material.

Supplementary file1 (PDF 622 KB)

## Abbreviations

AUC: Area under the receiver operating characteristic curve
MASLD: Metabolic dysfunction-associated steatotic liver disease
MRE: Magnetic resonance elastography
MRI: Magnetic resonance imaging
N: Newton
PDFF: Proton density fat fraction
QUS: Quantitative ultrasound
RF: Radiofrequency
ROI: Region of interest
SCD: Distance between liver capsule and liver surface
UGAP: Ultrasound-guided attenuation parameter
US: Ultrasound

## Declarations

### Guarantor

The scientific guarantor of this publication is Marie Byenfeldt.

### Statistics and biometry

One of the authors has significant statistical expertise.

### Study subjects or cohorts overlap

No study subjects or cohorts have been previously reported.

### Methodology

• prospective

• diagnostic and experimental study

• performed at one institution
