# Supplementary material for: Altered probe pressure and body position increase diagnostic accuracy for men and women in detecting hepatic steatosis using quantitative ultrasound
Source: Eur Radiol. 2024 Mar 8;34(9):5989–99. doi: 10.1007/s00330-024-10655-1 (PMC11364715; doi:10.1007/s00330-024-10655-1)
Supplement: Supplementary file 1 — Supplementary file1 (PDF 622 KB) [file 330_2024_10655_MOESM1_ESM.pdf]

Altered probe pressure and body position increases diagnostic accuracy for men and women in detecting hepatic steatosis using quantitative ultrasound

ELECTRONIC SUPPLEMENTARY MATERIAL

<u>Supplementary material:</u>

1. ***Method description of applied probe force measurements.***
2. ***Evaluation of UGAP diagnostic performance with single measurement in same spot of liver parenchyma.***
3. ***Evaluation of probe force with single measurement in same spot of liver parenchyma.***
4. ***Method description of magnetic resonance measurements.***
5. ***Results for probe force influence on skin-to-liver capsule distance.***

Supplementary material 1, Method description of applied probe force meaurements.

The applied probe force was measured using a custom-built probe adapter. In short, a steel metal plate with a wire strain gauge and ball bearing sled was attached to the holder of the C1-6 probe. A custom 3D-printed plastic probe holder shell was then attached to the sled with a contact point on the wire strain gauge. The wire strain gauge was connected in a Wheatstone bridge circuit, amplified by instrument amplifiers, and sampled by a microcomputer (Arduino mini pro). The setup allowed probe force to be measured along the direction of the probe. The measured force had a linear range of 0-250 N and was presented on a display with 1 N resolution. A pedal was also connected to the microcomputer and setup to allow both zero-reset (double tap) and locking of the measured force (single tap, Supplementary Figure 1).

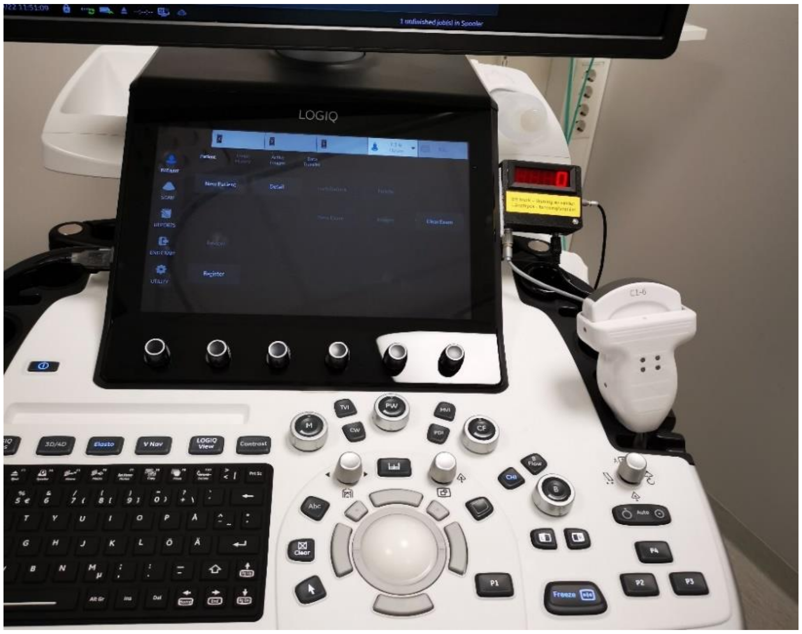

Supplementary Figure 1. Custom-built probe adapter with a plastic probe holder shell and the measured probe force presented on a display.

Supplementary material 2, Evaluation of UGAP diagnostic performance with single measurement in same spot of liver parenchyma.

As steatosis in early stages commonly has heterogeneous distribution in the liver, increased exactness for evaluation of the diagnostic performance of UGAP was achieved with a single estimation from MRI and US for each participant in the supine position. Data collection was performed by one radiologist and one radiographer/sonographer side-by-side with the MRI and US images on recall in the US device to ensure exactness of the comparison between methods, and estimations for both MRI and US were predominantly collected in liver segment 8. For UGAP (Supplementary Figure 2), no information was provided on the ROI size from the vendor, so we used an estimated UGAP ROI measure to determine the size of the PDFF ROI. Consequently, to match the size from one single measurement of UGAP performed with normal 4 N probe force, PDFF was estimated as Ø 35 mm (Supplementary Figure 3).

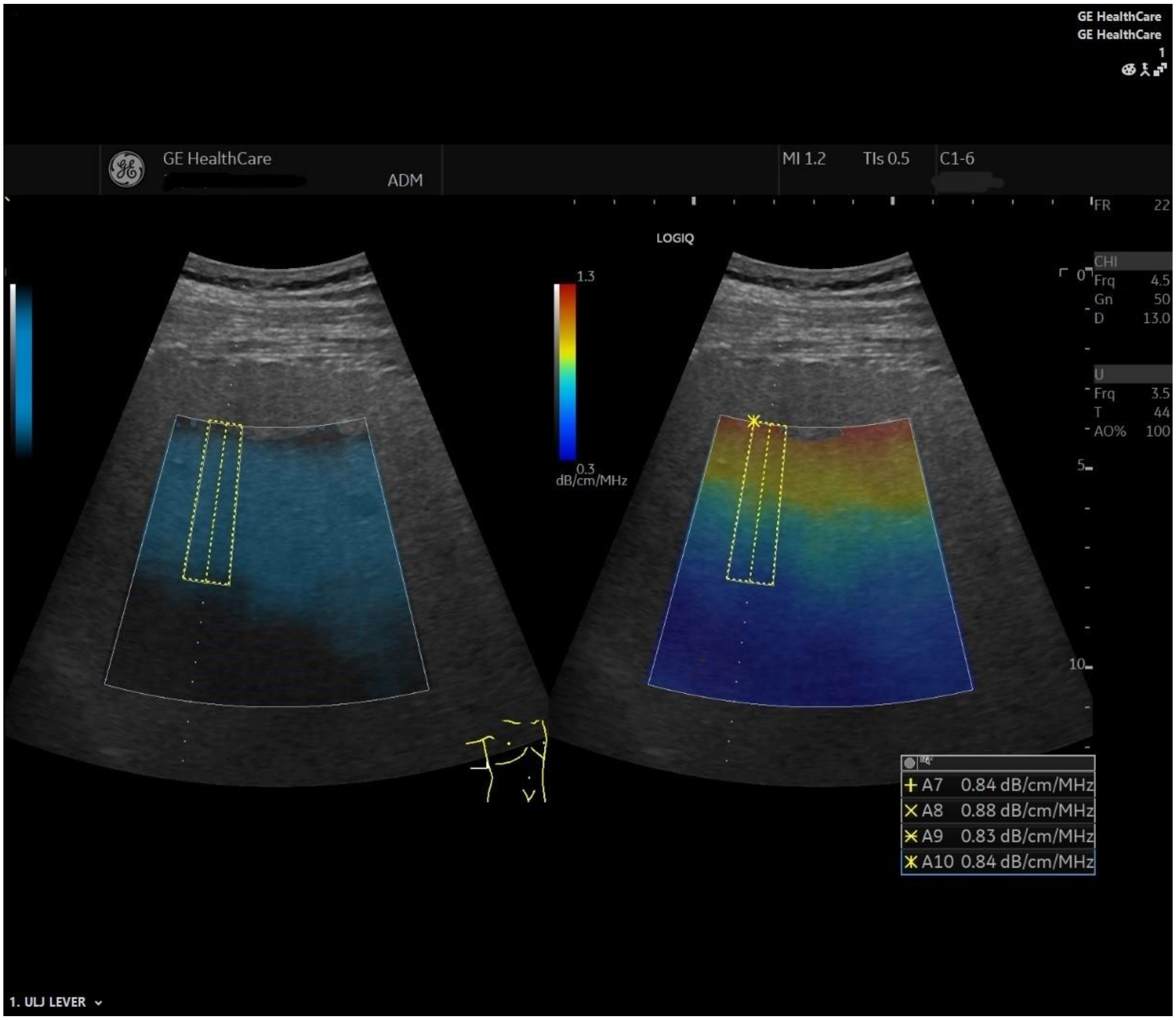

Supplementary Figure 2. The UGAP image shows a quality map (left) and an attenuation map (right) with a rectangular region of interest (ROI).

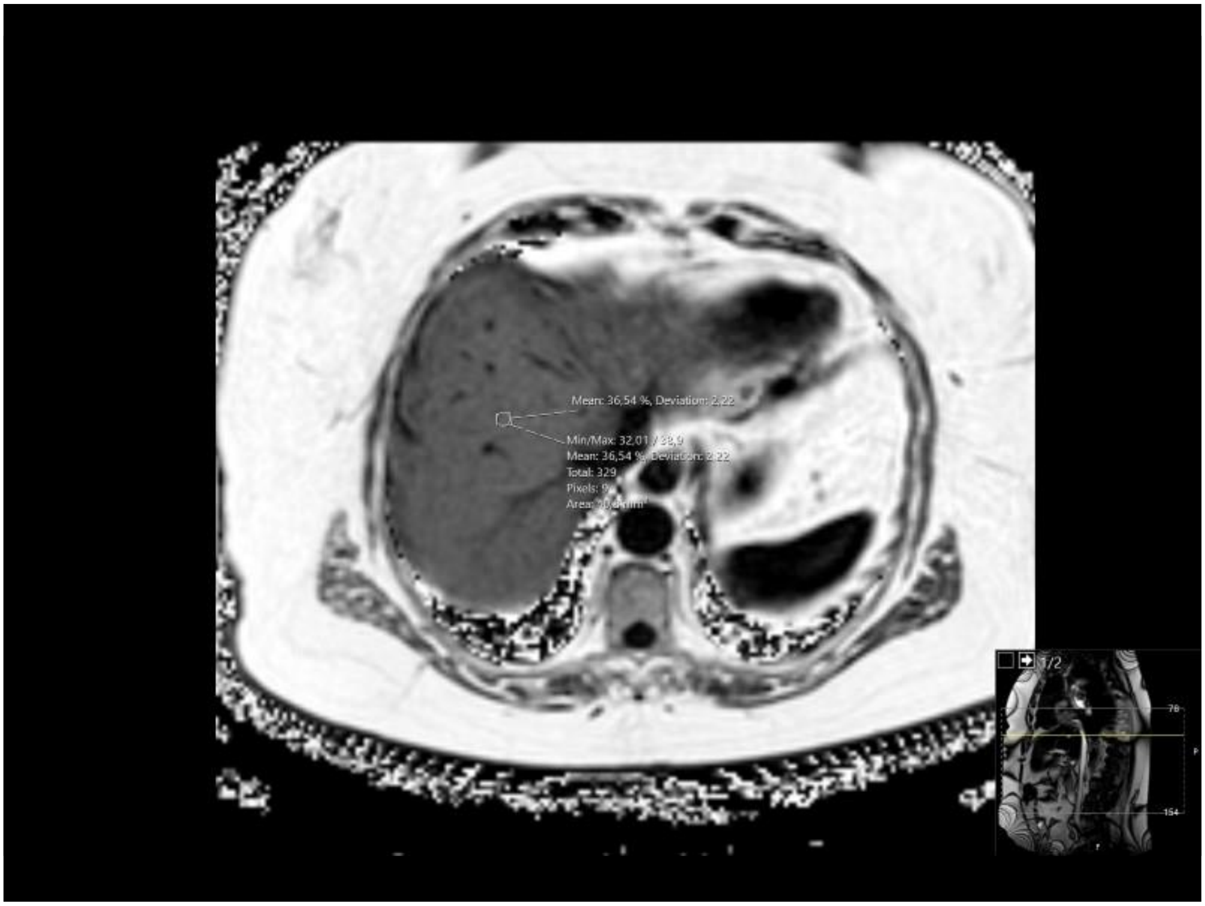

Supplementary Figure 3. One estimation for each participant in the supine position and predominantly in liver segment 8 with PDFF ROI Ø 35 mm to match the UGAP measurement size.

Results for PDFF showed no normal data distribution and was log10 transformed. The diagnostic performance of UGAP in this exact single measurement yielded an AUC of 0.95 (95% CI 0.91 – 1.00, Supplementary Figure 4).

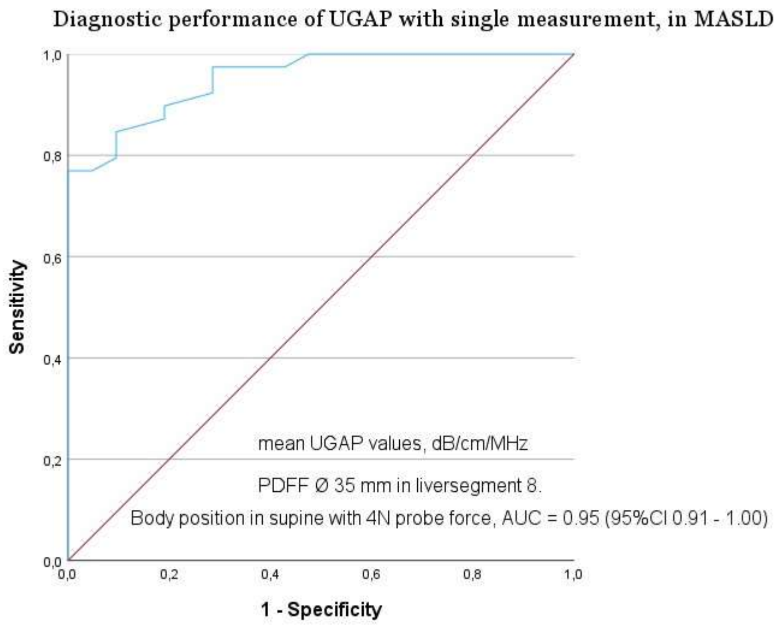

Supplementary Figure 4. Area under the curve (AUC) plots illustrate the diagnostic performance of ultrasound-guided attenuation parameter (UGAP) for body in supine with normal 4N probe force. AUC = 0.95(95% CI 0.91 – 1.00). For each participant, one UGAP measurement and one PDFF Ø 35 mm estimation was performed with the ROI position predominantly in liversegment 8. MRI-PDFF ≥ 5 % defined as the cut-off for steatosis grade S1. CI=confidence interval, MASLD= metabolic dysfunction-associated steatotic liver disease, MRI= magnetic resonance imaging, PDFF=proton density fat fraction.

Supplementary material 3, Evaluation of probe force with single measurement in same spot of liver parenchyma.

To increase the exactness of the investigation on the influence of probe force, data collection was performed with two measurements in the exact same ROI position in the liver parenchyma: a single measurement performed with normal 4 N probe force directly followed by a single measurement with increased 30 N probe force in both the supine and 30° left decubitus position. The mean UGAP values of these two different measurements were then compared.

The results showed a difference between normal and increased probe force for the mean UGAP values both in supine (0.65, SD 0.13 vs. 0.68, SD 0.12 dB/cm/MHz, $p<0.001$, N=60) and left decubitus (0.65, SD 0.13 vs. 0.69, SD 0.13 dB/cm/MHz, $p<0.001$, n=59).

Supplementary material 4, Method description of magnetic resonance measurements.

PDFF was acquired using a modified Dixon sequence with the following parameters: field-of-view (FoV), 400 x 350 mm^2; matrix size, 160 x 140 (reconstructed, 192); slice thickness, 6 mm; gradient multi-echo, TR, 5.7 ms; six echoes, the first TE 0.97 ms and with delta TE 0.7. Breath-hold time was 16 s. A fat fraction map was automatically generated by the vendor's software. The MRE data were acquired using the following parameters: FoV, 450 x 405 mm^2; matrix size, 76 x 66 (reconstructed, 320); slice thickness 10 mm; echo-planar imaging (EPI) single shot spin echo; TR, 1000 ms; TE, 54 ms and fat suppression. The acoustic transducer (Resoundant, Rochester, Minnesota, United States) was used with a frequency of 60 Hz, an amplitude adjusted for body weight, and single breath holds of 13 s. An elastogram map was generated by the vendor's software.

Supplementary material 5, Results for probe force influence on skin-to-liver capsule distance.

Normal 4 N vs. increased 30 N probe force in supine position yielded an skin-to-liver capsule distance (SCD) of 2.10 (range 0.94 – 3.19) cm vs. 1.88 (range 0.83 – 2.93) cm ($p<0.001$, N=60). For individuals in supine with the max distance, the SCD decreased from 3.19 to 2.93 cm with increased 30 N probe force.

For 30° left decubitus position, normal 4 N vs. increased 30 N probe force yielded an SCD of 2.02 (range 0.96 – 3.22) cm vs. 1.85 (range 0.83 – 3.03) cm ($p<0.001$, n=59). For individuals in left with max distance, the SCD decreased from 3.22 to 2.96 cm with increased 30 N probe force.
